# The Sex-Specific Detrimental Effect of Diabetes and Gender-Related Factors on Pre-admission Medication Adherence Among Patients Hospitalized for Ischemic Heart Disease: Insights From EVA Study

**DOI:** 10.3389/fendo.2019.00107

**Published:** 2019-02-25

**Authors:** Valeria Raparelli, Marco Proietti, Giulio Francesco Romiti, Andrea Lenzi, Stefania Basili, The EVA Collaborative Group, Claudio Tiberti

**Affiliations:** Author Affiliations: Department of Experimental Medicine, Medical Physiopathology, Food Science and Endocrinology Section, Sapienza University of Rome, Rome, Italy; Department of Cardiovascular, Respiratory, Nephrologic, Anaesthesiologic and Geriatric Sciences, Sapienza University of Rome, Rome, Italy; Department of Internal Medicine and Medical Specialties, Sapienza University of Rome, Rome, Italy; Department of Translational and Precision Medicine, Sapienza University of Rome, Rome, Italy; Emergency Department Stroke Unit, Sapienza University of Rome, Rome, Italy; McGill University Health Centre Research Institute, Centre for Outcomes Research and Evaluation, Montreal, QC, Canada; Department of Public Health and Infectious Disease, Sapienza University of Rome, Roma, Italy; San Raffaele Roma Open University and Inter-institutional Multidisciplinary Biobank, IRCCS San Raffaele Pisana, Rome, Italy; Department of Movement, Human and Health Sciences Section of Health Sciences, Unit of Endocrinology, Università degli Studi di Roma “Foro Italico”, Rome, Italy; Department of Biomedical and Specialty Surgical Sciences, University of Ferrara, Ferrara, Italy; Department of Medico-Surgical Sciences and Biotechnologies, Sapienza University of Rome, Latina, Italy; Department of AngioCardioNeurology, IRCCS NeuroMed, Pozzilli, Italy; Department of Radiological, Oncological and Pathological Sciences, Sapienza University of Rome, Rome, Italy; Chest Pain Unit, Policlinico Umberto I, Rome, Italy; Department of Emergency Medicine, Policlinico Umberto I, Sapienza University of Rome, Rome, Italy; Department of Surgical Sciences, Sapienza University of Rome, Rome, Italy; Nursing Team Catheterization Lab Policlinico Umberto I, Rome, Italy; ^1^Department of Experimental Medicine, Sapienza University of Rome, Rome, Italy; ^2^Centre for Outcomes Research and Evaluation, McGill University Health Centre Research Institute, Montreal, QC, Canada; ^3^Department of Internal Medicine and Medical Specialties, Sapienza University of Rome, Rome, Italy; ^4^Department of Neuroscience, Istituto di Ricerche Farmacologiche “Mario Negri” IRCCS, Milan, Italy

**Keywords:** sex, gender, diabetes, medication adherence, ischemic heart disease, personality traits, employment status

## Abstract

**Background:** Sex and gender-related factors have been under-investigated as relevant determinants of health outcomes across non-communicable chronic diseases. Poor medication adherence results in adverse clinical outcomes and sex differences have been reported among patients at high cardiovascular risk, such as diabetics. The effect of diabetes and gender-related factors on medication adherence among women and men at high risk for ischemic heart disease (IHD) has not yet been fully investigated.

**Aim:** To explore the role of sex, gender-related factors, and diabetes in pre-admission medication adherence among patients hospitalized for IHD.

**Materials and Methods:** Data were obtained from the Endocrine Vascular disease Approach (EVA) (ClinicalTrials.gov Identifier: NCT02737982), a prospective cohort of patients admitted for IHD. We selected patients with baseline information regarding the presence of diabetes, cardiovascular risk factors, and gender-related variables (i.e., gender identity, gender role, gender relations, institutionalized gender). Our primary outcome was the proportion of pre-admission medication adherence defined through a self-reported questionnaire. We performed a sex-stratified analysis of clinical and gender-related factors associated with pre-admission medication adherence.

**Results:** Two-hundred eighty patients admitted for IHD (35% women, mean age 70), were included. Around one-fourth of the patients were low-adherent to therapy before hospitalization, regardless of sex. Low-adherent patients were more likely diabetic (40%) and employed (40%). Sex-stratified analysis showed that low-adherent men were more likely to be employed (58 vs. 33%) and not primary earners (73 vs. 54%), with more masculine traits of personality, as compared with medium-high adherent men. Interestingly, women reporting medication low-adherence were similar for clinical and gender-related factors to those with medium-high adherence, except for diabetes (42 vs. 20%, *p* = 0.004). In a multivariate adjusted model only employed status was associated with poor medication adherence (OR 0.55, 95%CI 0.31–0.97). However, in the sex-stratified analysis, diabetes was independently associated with medication adherence only in women (OR 0.36; 95%CI 0.13–0.96), whereas a higher masculine BSRI was the only factor associated with medication adherence in men (OR 0.59, 95%CI 0.35–0.99).

**Conclusion:** Pre-admission medication adherence is common in patients hospitalized for IHD, regardless of sex. However, patient-related factors such as diabetes, employment, and personality traits are associated with adherence in a sex-specific manner.

## Introduction

Poor medication adherence to long-term pharmacological therapies is a public health concern that affects mostly patients with non-communicable chronic diseases (1, 2) such as type 2 diabetes mellitus and ischemic heart disease (IHD), both global epidemics of the twenty-first century for men and women (3–5). Among diabetic patients, the non-adherence to glucose-lowering drugs results in a suboptimal glycemic control with higher risk of diabetes-related microvascular complications (6, 7). Moreover, diabetic patients should be treated with cardiovascular drugs to prevent macrovascular complications including IHD (8–10). Sex differences in the cardiovascular consequences of diabetes exist and should be specifically addressed in the management of diabetic patients (11–13).

Multiple variables affecting physicians and patients contribute to medication non-adherence, which in turn negatively affects treatment outcomes and causes psychosocial complications, reduces patients' quality of life, and wastes health care resources (14–16). The World Health Organization suggests that improving adherence requires a multidimensional approach (i.e., social and economic factors, health-care team and system-related factors, condition-related factors, therapy-related factors, and patient-related factors), highlighting that psycho-socio-cultural factors need to be considered as contributors (17).

Even though sex- (i.e., biological factors) and gender- (i.e., psycho-socio-cultural factors) differences play a very important role in influencing clinical outcomes, both aspects are generally overlooked and under-reported (18, 19). While biological sex variables refer to objectively measurable organs, hormones, genes, anatomy, and physiology, gender represents a social construct that is linked to economic and social status (20). It has several dimensions, including gender identity (how individuals perceive and present themselves), gender norms (behavioral expectations based on individual sex including role in the family, in the workplace, in society, etc.), and gender relations (emotional and economic relations between individuals) (21). As sex and gender are not independent, exclusively assessing one or the other fails to account for identified variations in health (22, 23). Specifically, their role and interaction as determinants of medication adherence are still not fully understood. Moreover, the complexity of gender and the lack of a validated standardized measurement, with the exception of the GENESIS-PRAXY gender score (23, 24), both contribute to the paucity of data available including medication adherence.

Based on a prospective cohort of patients hospitalized for IHD events, with the unique systematic collection of gender-related variables, we explored the factors associated with pre-admission medication adherence and whether any sex differences exist in the effect of diabetes and gender-related variables on pre-admission medication adherence.

## Materials and Methods

Data for the present analysis come from the “Endocrine Vascular disease Approach” (EVA) project (ClinicalTrials.gov Identifier: NCT02737982), an ongoing prospective observational study aiming to explore sex and gender differences in the interaction of platelet function, sex hormone balance, and coronary microvascular dysfunction in IHD (25). In brief, EVA is a registry of men and women, aged 18 and older, who were referred to the cardiac catheterization laboratory to undergo coronary angiography and/or percutaneous coronary intervention for suspected IHD, Patients with active cancer (i.e., currently treated with chemotherapy or at ≤5 years from diagnosis), current pregnancy, previous coronary artery bypass graft, documented moderate-severe valvular heart disease, and prosthetic valve carriers were excluded. Therefore, the EVA study population includes patients with stable coronary artery disease, patients with non-ST elevation acute coronary syndrome and patients with ST elevation myocardial infarction. The recruitment phase is still ongoing.

The study was conducted in full conformance with the principles of the Declaration of Helsinki, the laws and regulations of Italy, or whichever afforded greater protection to the individual. The study obtained the required authorization by the local Ethics Committee of Policlinico Umberto I, Sapienza University of Rome (reference 3786, 24/09/2015). Written informed consent has been obtained from all patients.

Among the study population, we selected baseline data from patients recruited until August 2018.

### Baseline Clinical Characteristics and Gender-Related Factors

We selected patients with complete information at baseline regarding: (1) anthropometric data (including height, weight, blood pressure, heart rate); (2) independent functional status assessed by the Duke Activity Status Index (26); (3) presence of diabetes; (4) cardiovascular risk factors including family history of cardiovascular disease; hypertension; dyslipidemia; chronic kidney disease; previous cardiovascular events including prior MI and stent implantation. Patients' baseline characteristics were selected from a combination of medical record abstraction and standardized in-person interviews administrated by trained personnel.

According to the definition of gender components designed by the Women's Health Research Network (21), we collected the following gender-related factors: (1) gender role: household's primary earner status and employment status; (2) gender relations: marital status (i.e., married/living with partner vs. others); (3) gender identity: Bem Sex Role Inventory (BSRI) masculine and feminine (27), and level of stress (defined through the 10-item perceived stress scale) (28); (4) institutionalized gender: low socioeconomic status [defined as low education level (i.e., less than secondary school) and/or low household income (i.e., <1,000 Euro per month)]. Furthermore, we considered the following as risk-taking behaviors: alcohol consumption (i.e., more than 2 units per day for women and 3 units per day for men), physical inactivity (defined as no recreational activity or less than once per week), and smoking habits (current smoker). All the gender-related variables were collected through self-reported questionnaires.

### Pre-admission Medication Adherence Assessment

Pre-admission medication adherence has been assessed using a self-administrated questionnaire at the baseline visit. A simple self-reported measure of medication adherence, which has been shown to be predictive of blood pressure control (29), was used to measure medication-taking behavior (i.e., trouble in remembering to take medication, spontaneous interruption due to personal judgment) among our hospitalized patients undergoing coronary angiography for suspected IHD. Polypharmacy was defined as more than 5 drugs assumed daily by the patient as previously reported (30). Information concerning the class of drugs taken by the patient include: antithrombotic agents (i.e., anticoagulants and antiplatelet drugs), b-adrenergic receptor blockers (b-blockers), hydroxylmethylglutaryl-coenzyme A reductase inhibitors (statins), angiotensin-converting enzyme inhibitors, angiotensin II receptor blockers, oral glucose lowering drugs, or subcutaneous insulin.

### Statistical Analysis

After verification of non-normality, continuous variables were reported as median and interquartile range (IQR). Differences between the groups (low-adherent vs. medium-high adherent) were established, according to non-normal distribution, with a non-parametric test, the Mann-Whitney *U*-test. Categorical variables were reported as counts and percentages, and differences between groups (low-adherent vs. medium-high adherent) were evaluated with chi-square test or Fisher's Exact test when appropriate (cell count <5).

A logistic regression model was computed to identify the predictors of medium-high adherence. Both in the overall model, as well as in the sex-stratified analysis, variables which significantly differed at baseline with a *p*-value < 0.10 were identified to be included in a univariate analysis. All variables associated with adherence for <10% (*p* < 0.10) at univariate analysis, were included in the final multivariate model. A two-sided *p*-value < 0.05 was considered as statistically significant. All analyses were performed using SPSS v. 25.0 (IBM, NY, USA).

## Results

Among the EVA cohort, 280 (median [IQR] age 70 [63–76] years; 35% women) patients hospitalized for IHD were analyzed. Preadmission low-adherence was reported in 24% (*n* = 67) of patients, regardless of sex.

Clinical characteristics according to adherence are reported in Table 1. Low-adherent patients were more likely to be diabetic as compared with adherent patients. No statistically significant difference in pre-admission medication adherence was found between women and men with or without prior history of IHD (74 vs. 77%, respectively). No differences in age, sex, blood pressure, body mass index, main cardiovascular co-morbidities, type of drugs, and polypharmacy were observed between medium-high adherent and low-adherent subjects. The distribution of gender-related factors according to pre-admission medication adherence status is shown in Table 2. The only gender-related factor statistically more prevalent in low-adherent patients was to be employed. Other psycho-socio-cultural factors were similar among the 2 groups.

**Table 1:** Baseline clinical characteristics of EVA patients according to pre-admission medication adherence.

	**Low adherence (*n* = 67)**	**Medium-high adherence (*n* = 213)**	***p***
Age, *years* median [IQR]	68 [61–78]	71 [63–76]	0.447
Women, n (%)	24 (35.8)	74 (34.7)	0.872
BMI, *kg/m^2^* median [IQR]	27.0 [24.8–30.0]	26.5 [24.2–29.5]	0.447
SBP, *mmHg* median [IQR]	130 [120–150]	130 [120–140]	0.104
DBP, *mmHg* median [IQR]	80 [70–85]	80 [70–80]	0.537
HR, *bpm* median [IQR]	70 [62–80]	70 [60–76]	0.239
Hypertension	57 (86.4)	181 (85.0)	0.781
Dyslipidemia	41 (61.2)	122 (57.3)	0.571
Type 2 diabetes	27 (40.3)	58 (27.2)	0.042
Active Smoke	15 (22.7)	49 (23.2)	0.934
Alcohol Abuse	11 (16.4)	36 (17.7)	0.805
Physical inactivity	57 (85.1)	167 (78.4)	0.234
History of CAD	28 (41.8)	79 (37.1)	0.490
Vascular disease	21 (31.8)	58 (27.2)	0.470
Stroke/TIA	7 (10.6)	24 (11.3)	0.881
CKD	17 (26.2)	53 (25.7)	0.945
Polypharmacy	38 (56.7)	106 (49.8)	0.321
DASI, median [IQR]	27.0 [19.0–42.7]	32.2 [19.0–49.8]	0.153
**Therapy pre-admission**			
- Glucose lowering drugs	17 (25.4)	41 (19.2)	0.281
- Insulin	7 (10.4)	12 (5.6)	0.172
- Statins	32 (47.8)	110 (51.6)	0.579
- Antiplatelets	43 (64.2)	135 (63.4)	0.906
- Anticoagulants	8 (11.9)	19 (8.9)	0.465
- Beta-Blockers	30 (44.8)	96 (45.1)	0.966
- ACEi	19 (28.4)	57 (26.8)	0.798
- ARBs	18 (26.9)	76 (35.7)	0.183

*Data are presented as number of patients (%) unless otherwise specified*.

*IQR, interquartile range; BMI, body mass index; SBP, systolic blood pressure; DBP, diastolic blood pressure; HR, heart rate; CAD, coronary artery disease; TIA, Transient ischemic attack; CKD, chronic kidney disease; DASI, Duke Activity Status Index; ACEi, Angiotensin converting enzyme inhibitors; ARBs, Angiotensin II receptor blockers. When missing data are more than 10%, they are reported in brackets*.

**Table 2:** Gender-related factors of EVA patients according to pre-admission medication adherence.

	**Low adherence (*n* = 67)**	**Medium-High Adherence (*n* = 213)**	***p***
Employed status	27 (40.3)	57 (27.0)	0.039
Primary earner^#^	19 (38.0)	83 (49.4)	0.156
Male BSRI, median [IQR]	5.0 [4.1–5.6]	4.7 [4.1–5.4]	0.552
Female BSRI, median [IQR]	5.8 [5.2–6.4]	5.9 [5.3–6.2]	0.991
High Stress at home^#^	14 (29.2)	44 (26.2)	0.682
Married/living with partner	46 (68.7)	149 (70.0)	0.840
Low SES^*^	36 (53.7)	96 (45.1)	0.215

*Data are presented as number of patients (%) unless otherwise specified*.

SES, socioeconomic status; BSRI, Bem Sex Role Inventory;

* *SES was defined based on low education level and/or personal income*.

# *data available for 215 patients*.

When data were stratified by sex, we observed that low-adherent men were most likely employed yet not primary earners, and with more masculine personality traits (Table 3). On the other hand, low-adherent women were most likely diabetic and physically inactive, with no statistical differences in any gender-related factor (Table 4). No differences in the types of drugs taken were observed in both sexes.

**Table 3:** Clinical and gender-related factors in men according to pre-admission medication adherence.

	**Low adherence (*n* = 43)**	**Medium-high adherence (*n* = 139)**	***p***
Age, *years* median [IQR]	65 [60–74]	69 [63–75]	0.157
BMI, *kg/m^2^* median [IQR]	27.5 [25.3–31.0]	27.1 [25.1–29.4]	0.576
SBP, *mmHg* median [IQR]	120 [110–130]	120 [110–130]	0.200
DBP, *mmHg* median [IQR]	80 [70–85]	80 [70–85]	0.973
HR, *bpm* median [IQR]	70 [62–78]	69 [60–76]	0.279
Hypertension	37 (88.1)	123 (88.5)	0.944
Dyslipidemia	27 (62.8)	85 (61.2)	0.847
Type 2 diabetes	17 (39.5)	43 (30.9)	0.294
Active Smoke	13 (30.2)	36 (26.1)	0.593
Alcohol Abuse	10 (23.3)	32 (24.2)	0.895
Physical inactivity	33 (76.7)	104 (74.8)	0.798
History of CAD	23 (53.5)	61 (43.9)	0.270
Vascular disease	13 (31.0)	43 (30.9)	0.998
Stroke/TIA	4 (9.5)	14 (10.1)	0.917
CKD	9 (22.0)	32 (23.7)	0.816
Polypharmacy	25 (58.1)	72 (51.8)	0.466
DASI, median [IQR]	8.5 [3.0–14.0]	5.0 [2.0–11.5]	0.610
Employed status	25 (58.1)	46 (33.3)	0.004
Primary earner^#^	18 (54.5)	77 (73.3)	0.042
Male BSRI, median [IQR]	5.2 [5.0–5.8]	4.9 [4.4–5.5]	0.059
Female BSRI, median [IQR]	6.0 [5.1–6.4]	5.6 [5.−6.2]	0.581
High stress at home^#^	5 (18.8)	19 (18.4)	0.969
Married/living with partner	33 (76.7)	109 (78.4)	0.817
Low SES^*^	20 (46.5)	54 (38.8)	0.371

*Data are presented as number of patients (%) unless otherwise specified*.

IQR, interquartile range; BMI, body mass index; SBP, systolic blood pressure; DBP, diastolic blood pressure; HR, heart rate; CAD, coronary artery disease; TIA, Transient ischemic attack; CKD, chronic kidney disease; DASI, Duke Activity Status Index; SES socioeconomic status; BSRI, Bem Sex Role Inventory;

* *SES was defined based on low education level and/or personal income*.

# *data available for 135 men*.

**Table 4:** Clinical and gender-related factors in women according to pre-admission medication adherence.

	**Low adherence (*n* = 24)**	**Medium-high adherence (*n* = 74)**	***p***
Age, years median [IQR]	72 [64–81]	74 [65–78]	0.568
BMI, kg/m^2^ median [IQR]	26.3 [24.2–28.8]	24.9 [22.2–29.7]	0.435
SBP, mmHg median [IQR]	130 [125–140]	130 [120–140]	0.315
DBP, mmHg median [IQR]	75 [70–90]	70 [70–80]	0.259
HR, bpm median [IQR]	72 [60–80]	72 [65–77]	0.526
Hypertension	20 (83.3)	58 (78.4)	0.601
Dyslipidemia	14 (58.3)	37 (50.0)	0.478
Type 2 diabetes,	10 (41.7)	15 (20.3)	0.037
Active Smoke	2 (8.7)	13 (17.8)	0.294
Alcohol Abuse	1 (4.2)	4 (5.6)	0.781
Physical inactivity	24 (100.0)	63 (85.1)	0.045
History of CAD	5 (20.8)	18 (24.3)	0.726
Vascular disease	8 (33.3)	15 (20.3)	0.189
Stroke/TIA	3 (12.5)	10 (13.5)	0.899
CKD	8 (33.3)	21 (29.6)	0.730
Polypharmacy	13 (54.2)	34 (45.9)	0.484
DASI, median [IQR]	11 [7–25]	10 [6.8–15]	0.114
Employed status	2 (8.3)	11 (15.1)	0.401
Primary earner^#^	1 (5.9)	6 (9.5)	0.637
Male BSRI, median [IQR]	4.3 [3.7–4.6]	4.6 [4.0–5.4]	0.106
Female BSRI, median [IQR]	5.7 [5.0–6.8]	6.0 [5.6–6.6]	0.773
High stress at home^#^	8 (50.0)	25 (38.5)	0.400
Married/living with partner	13 (54.2)	40 (54.1)	0.992
Low SES^*^	16 (66.7)	42 (56.8)	0.391

*Data are presented as number of patients (%) unless otherwise specified*.

IQR, interquartile range; BMI, body mass index; SBP, systolic blood pressure; DBP, diastolic blood pressure; HR, heart rate; CAD, coronary artery disease; TIA, Transient ischemic attack; CKD, chronic kidney disease; DASI, Duke Activity Status Index; SES socioeconomic status; BSRI, Bem Sex Role Inventory;

* *SES was defined based on low education level and/or personal income*.

# *data available for 80 women*.

Therefore, we explored the determinants of pre-admission adherence: only being employed was independently associated with medication adherence status [Odds Ratio (OR) 0.55, 95% Confidence Interval (CI) 0.31–0.97, *p* = 0.041] in our cohort (Table 5). However, when we stratified the analysis by sex, only a BSRI masculine (OR 0.59, 95%CI 0.35–0.99, *p* = 0.048) was inversely associated with adherence in men, while only diabetes (OR 0.36; 95%CI 0.13–0.96, *p* = 0.041) in women (Table 5).

**Table 5:** Multivariate logistic regression of pre-admission medication adherence in patients admitted for ischemic heart disease.

	**OR**	**95% CI**	***p***
**OVERALL**			
Employed status	0.55	0.31–0.97	0.041
**MALE PATIENTS**			
Male BSRI	0.59	0.35–0.99	0.048
**FEMALE PATIENTS**			
Type 2 diabetes	0.36	0.13–0.96	0.041

*OR, Odds Ratio; CI, confidence interval; BSRI, Bem Sex Role Inventory*.

## Discussion

The main findings of our analysis are that: (i) pre-admission poor adherence was highly prevalent, characterizing more than one fourth of men and women hospitalized for IHD; (ii) diabetes was the only cardiovascular risk factor significantly prevalent in low-adherent patients with IHD before hospitalization; (iii) among the gender-related variables, employed status, as proxy of the gender role, was negatively associated with medication adherence; and (iv) factors related to pre-admission adherence differed between sexes, with diabetes being more relevant for women, while masculine traits of personality were independently associated with low-adherence in men.

Medication low-adherence is a major and costly obstacle in the management of common non-communicable chronic diseases, such as hypertension and diabetes (16, 31, 32). The prevalence of medication non-adherence varies tremendously depending on the population studied, the method used for assessment of medication use (e.g., self-report or pharmacy refill data) and the specific medications assessed (15, 33–35). Moreover, significant sex differences were previously reported in general drug-users in terms of intensity of medication use and compliance to treatment (36). Interestingly, in our cohort of patients admitted for an IHD event, the pre-admission poor medication adherence was similarly high (25%) among women and men. However, we identified different factors associated to inappropriate drug behavior among sexes that should be considered when targeting obstacles for improving patient adherence to pharmacological treatment.

Diabetes is the only condition that reverses the female advantage in cardiovascular risk (37). Diabetic women experience worse clinical outcomes compared to age-matched diabetic men, especially in the setting of ischemic heart disease (38). In our cohort, diabetic women hospitalized for an ischemic coronary event were more likely low-adherent to therapy before admission. Diabetes was found as the only clinical determinant of pre-admission medication adherence in a sex-specific manner, despite the low sample of women enrolled in the present analysis. On the other hand, diabetes loses its explanatory power when gender-related factors are considered in the subgroup of male patients. We provide an example of how controlling and adjusting the analysis for sex can lead to a misinterpretation of data, especially when the women are underrepresented in a study population. Therefore, an analysis disaggregated by sex might inform our better understanding of the outcomes of interest in men and women. In fact, the approach of reporting data disaggregated by sex and a sex-stratified analysis are recommended by the international clinical research stakeholders (39, 40).

The distinction between sex and gender, which is clear and common in the social sciences, has largely been neglected in health science. In truth, sex and gender are often erroneously used and/or measured interchangeably (18). The integration of sex- and gender-based analysis is a much-needed and, yet, uncommon approach to improving the quality of evidence and guaranteeing the generalizability of findings. However, when considering gender in the evaluation of clinical outcomes, the first hurdle for researchers originates from the apparent lack of standardized methods to measure the complexities of all that gender encompasses. Recently, through a Pan-Canadian collaboration of a multi-disciplinary team of scientists, a comprehensive list of gender-related variables was established and collected in the setting of premature cardiovascular disease (23). Using the EVA cohort, we could now potentially describe every dimension of gender through a granularity of data which is rarely available. In the analysis adjusted for sex, employment status was the only factor associated to medication adherence: specifically, employed status has a negative influence on adherence to therapy. This difference in adherence between unemployed and employed could be attributed to the busy work schedules of working patients, which could interfere with adequate behaviors of self-care. However, in literature, the link between employed status and medication adherence has been barely explored. Our findings have therefore raised important unexplored questions on the mechanisms explaining the association between gender roles and medication adherence in patients at high cardiovascular risk. Apart from work status, personality traits (considered as expressions of gender identity) seem to play a pivotal role in medication adherence (41, 42). It was reported that some personality traits such as emotional stability, interpersonal orientation, and motivation in goal-directed behavior are associated to medication adherence in individuals with chronic disease (41). In this light, we observed that beyond clinical characteristics, a higher BSRI masculine score, calculated on personality traits (including risk-taking behavior, independence, competitiveness, strong personality, etc.) can be negatively associated to pre-admission medication adherence in men hospitalized for IHD. This finding may be explained by the tendency of male patients with these personality traits to put high trust in their own ability to manage their health, therefore underestimating the importance of adherence to health providers' prescriptions (41, 43).

The present analysis has several strengths worth mentioning. We were uniquely positioned to evaluate all 4 dimensions that gender constructs encompass in a “real-world,” contemporary cohort of patients with IHD. We also had a granularity in the data exploring gender constructs which is largely uncommon. Finally, as recommended by international societies and funding agencies (39, 40), we could report the data disaggregated by sex.

### Limitations

Some important limitations of the present analysis must be stated. As any observational cohort study, some confounders might not have been assessed and could influence our final multivariate model, as could any missing data points.

Furthermore, the sample size is small, and the EVA study was not specifically designed to test the effect of diabetes on pre-admission medication adherence outcomes. The lack of statistical significance in the effect of sex or gender-related factors may be due to the lack of power to address specific differences. We cannot exclude that, as the sample increases, some relevant differences might emerge. Moreover, the findings refer to a selected cohort of patients at very high cardiovascular risk, hospitalized for an ischemic event, at a single center in Italy, limiting the generalizability of our results. We cannot account for countries with specific differences in psychosocial and cultural factors.

Finally, medication adherence is related to the global patient assumption of drugs pre-admission, yet we cannot describe the nuances of adherence separately for each class of drugs, such as glucose lowering drugs or cardiovascular preventive drugs (i.e., anti-platelets or statins).

### Future Directions

Overall, the present analysis highlights the importance of considering the different effects of clinical characteristics (e.g., diabetes) and gender-related factors (e.g., personality traits or employment status) among women and men, in order to better identify and target high-risk subgroups of patients. The impact of pre-admission medication adherence on clinical outcomes in IHD patients, including diabetics, is a surprisingly underestimated issue (13) which requires further investigation (44, 45). Clinicians should consider the use of prior medication adherence as an indicator of future medication-taking behavior.

Future studies—powered and able to apply a gender transformative framework (46) in the consideration of the specific research questions—are warranted, with the overarching aim to ultimately design effective interventions to promote health and well-being.

New approaches such as big data analysis (47) across patient data registries across countries could be interesting avenues to explore the impact of gender-related factors as determinants of clinically relevant outcomes on a larger, population-based scale.

## Conclusion

Poor medication adherence is still a highly prevalent condition in patients at high cardiovascular risk. Medication adherence recognizes disease-centered and patient-centered determinants. Among them, gender-related factors (i.e., personality traits and gender role) and diabetes are associated in a sex-specific manner with pre-admission medication adherence of patients hospitalized for IHD. Future clinical trials should test if sex-specific and gender-sensitive interventions might improve medication adherence in high cardiovascular risk conditions, such as women with diabetes.

## Author Contributions

VR: study conception and design, coordination of multidisciplinary team research, patients' recruitment, interpretation, and writing manuscript; MP: study design, statistical analysis, data interpretation and writing manuscript; GR: database management, statistical analysis, and writing manuscript; AL: data interpretation, revision of the manuscript; SB: study conception and design, data interpretation, revision of the manuscript; EVA Collaborators: patients recruitment, data entry.

### Conflict of Interest Statement

The authors declare that the research was conducted in the absence of any commercial or financial relationships that could be construed as a potential conflict of interest.
